# Metacognition-Associated Factors in Physical and Occupational Therapy Students: A Cross-Sectional Study

**DOI:** 10.3390/brainsci14101041

**Published:** 2024-10-21

**Authors:** Keisuke Taniguchi, Naoki Maki, Harumi Sakamoto, Tomonari Inuta, Bokun Kim, Sechang Oh, Thomas Mayers

**Affiliations:** 1 Department of Rehabilitation, R Professional University of Rehabilitation, 2-12-31 Kawaguchi, Tsuchiura 300-3253, Japan; taniguchi@a-ru.ac.jp (K.T.); sakamoto@a-ru.ac.jp (H.S.); o-sechan@u.a-ru.ac.jp (S.O.); 2 Department of Physical Therapy, R Medical Care and Welfare Professional College, 2-12-31 Kawaguchi, Tsuchiura 300-3253, Japan; inuta@a-ru.ac.jp; 3 Future Convergence Research Institute, Changwon National University, 20 Changwondaehak-ro Uichang-gu, Changwon-si, 51140, Gyeongsangnam-do, Republic of Korea; fabulousbobo79@gmail.com; 4 Medical English Communications Center, Institute of Medicine, University of Tsukuba, 1-1-1 Tennodai, Tsukuba 305-8575, Japan; mayers@md.tsukuba.ac.jp

**Keywords:** metacognition, personality characteristics, mental health, physical and occupational therapy education, COVID-19

## Abstract

Background/Objectives: Supporting the mental well-being of students through their educational journey is of vital importance. The objective of this study was to investigate the relationship between metacognition, personality traits, and various factors affecting mental health in a cohort of physical therapy (PT) and occupational therapy (OT) students. Methods: This cross-sectional observational study involved a self-administered questionnaire (distributed in October to November 2020) that gathered demographic information and included six scales measuring personality characteristics, health literacy, self-management skills, exercise habits (benefits and barriers), cognitive thinking, and cognitive deliberateness and impulsivity. Results: A cohort of 195 PT and OT students participated in the study. The findings revealed significant associations between metacognition and personality, particularly the association between low cognitive self-confidence and high emotional instability. This link suggests that students who struggle with cognitive self-confidence may also experience greater emotional instability, highlighting a need for targeted mental health support. Additionally, health literacy was negatively correlated with anxiety, while metacognition was positively correlated with perceived exercise benefits. Conclusions: This study highlights the importance of individualized metacognitive approaches to support students’ mental well-being. Interventions should focus on strengthening cognitive self-confidence through methods such as resilience training, cognitive restructuring, and mindfulness practices to help manage emotional instability. Furthermore, promoting physical activity, particularly among female students, and incorporating gender-specific strategies could enhance mental health outcomes.

## 1. Introduction

In recent years, the prevalence of mental illness in Japan has been gradually increasing, and its economic impact has been reported to be very significant [1]. According to the Organisation for Economic Co-operation and Development’s (OECD’s) International Survey on Mental Health, the percentage of people with depression in Japan doubled from 7.9% in the 2013 survey to 17.3% in 2020 after the COVID-19 pandemic [2]. College students are said to be at the prime age for mental illness [3], and the exacerbation of mental health problems has a significant impact on student life [4]. Students in healthcare-related fields are perhaps the most vulnerable student population to mental illness, showing significantly higher rates of mood and anxiety disorders, psychological distress, and even suicidal ideation compared to the general student population [5,6]. Physical therapy (PT) and occupational therapy (OT) students are no exception; a 2022 study of PT and OT students in Canada showed higher levels of stress, anxiety, and/or depression compared to general-population samples [7].

Early detection and prevention measures to address mental illness are vital. In Japan, many universities have set up counseling services to try to tackle this issue, but with reports that depressive symptoms worsened under the influence of the COVID-19 pandemic [8,9], more needs to be done to understand and address this pressing situation. Risk factors for mental illness include loneliness and financial insecurity, while protective factors reportedly include the presence of someone to consult with, social support, exercise, and communication with others using social networking sites [10,11,12]. However, there is a paucity of research that examines these social environmental factors from the perspective of individual personality characteristics.

Personality characteristics and their association with metacognition have increasingly been the focus of attention in intervention methods for addressing depression and anxiety and improving positive mental health [13,14]. Research has suggested that metacognition and personality are often intertwined, with specific aspects of metacognition linked to certain personality traits, such as self-regulation, conscientiousness, cognitive confidence, and emotional stability [13,14,15]. Notably, metacognitive dysfunctions are also associated with personality disorders [16,17]. Personality characteristics are known to influence a person’s physical and mental behavior [18,19], and interventions that approach behavior change from the perspective of personality characteristics have been attempted [20]. Furthermore, the important association between health literacy and personality characteristics has also been reported [21,22,23].

Metacognition, which is defined as the brain’s ability to understand and control what it perceives, i.e., “thinking about thinking” [24,25], is essential for educational success and mental resilience, which is particularly important for students who are preparing to enter demanding healthcare fields. Moreover, metacognition has been reported to have an influence on depression through pathways related to anxiety [26,27,28]. Theoretically, metacognition comprises two main components: metacognitive knowledge, which includes awareness of one’s own cognitive abilities, understanding of various learning strategies, and knowledge of when and how to apply these strategies effectively; and metacognitive regulation, which refers to the ability to plan, monitor, and evaluate one’s cognitive processes [29,30]. Both components are vital for self-directed learning and personal growth. In practice, metacognitive skills enable students to adapt their learning strategies to meet academic demands while supporting their mental health by promoting more effective stress management and coping strategies [31,32]. Importantly, metacognition can be reliably assessed in both clinical and non-clinical populations using a variety of validated tools, allowing for precise measurement of its impact on mental health and learning [33].

A deeper understanding of metacognition and tailored interventions based on personality traits might help to address the increase in mental health disorders among students. Particularly relevant to the current study, metacognition has also been reported to contribute to learning and growth, especially among medical students, and is considered to be an essential skill for the development of medical care professionals working under stress [34,35,36,37]. While the concept of metacognition has become more widely known, the factors that constitute metacognition and the association between metacognition and personality traits have not been fully clarified. The purpose of this study, therefore, was to investigate the relationship between metacognition and personality characteristics among PT and OT students, with the hope that this knowledge would help support their mental health and, thereby, improve their educational experience, especially in the wake of the COVID-19 lockdowns.

## 2. Materials and Methods

### 2.1. Participants

The study participants were drawn from a convenience sample of 236 Japanese OT and PT students aged between 18 and 25 years of age, who were attending a private specialized medical school for physical and occupational therapy training. Exclusion criteria were (1) students who were aged 26 years or older and had previous work history and (2) students who did not give consent to participate.

All participants were fully informed in writing and orally in advance of the content and purpose of the research; the protection of personal information; and the fact that they would not suffer any disadvantages due to refusal, withdrawal, or discontinuation of participation. Written informed consent was obtained from all included participants. Approval for this study was granted by the Research Ethics Review Committee of Ibaraki Prefectural University of Health Sciences (Approval No. 932).

### 2.2. Study Design, Survey, and Instruments

This study used a cross-sectional observational design involving a self-administered questionnaire. The survey was carried out from October to November 2020, at the height of the COVID-19 pandemic. After a semester of online classes, students had returned to campus, but classes were also offered online in a hybridized fashion. The surveys were distributed on campus in paper form.

Participant demographic characteristics and metacognition were used as the main outcomes. A comprehensive approach was taken to assess various factors that could influence student mental health and well-being. To determine possible associations, six scales measuring personality characteristics, health literacy, self-management skills, exercise habits (benefits and barriers), cognitive thinking, and cognitive deliberateness and impulsivity were employed to provide detailed insights into how these diverse factors potentially impact on students’ overall cognitive and emotional functioning.

#### 2.2.1. Metacognition

The metacognitive characteristics of the study participants were assessed using a well-established measurement tool. The Japanese version of the Metacognitive Questionnaire (MCQ-30) [38] is an abbreviated 30-item version [39] of the original 65-item instrument [40]. The MCQ-30 measures metacognition over five subscales: (1) cognitive confidence (assessing confidence in attention and memory); (2) positive beliefs (assessing one’s positive beliefs about worries); (3) cognitive self-consciousness (assessing a tendency to focus attention on thought processes), (4) uncontrollability and danger (assessing negative beliefs about uncontrollability and danger); and (5) need to control thoughts (assessing negative beliefs regarding the consequences of not controlling one’s thoughts) [39]. Responses to the items are made using a 4-point Likert scale (English version: 1 = do not agree‚ 2 = agree slightly‚ 3 = agree moderately‚ and 4 = agree very much). The reliability (α = 0.72–0.87) of the Japanese version of the MCQ-30 has been confirmed among a group of 135 university students using the test–retest method [38].

#### 2.2.2. Personality Characteristics

To capture a comprehensive view of participants’ personality traits, an established assessment tool, the Big Five Scale, was employed [41]. This Japanese scale consists of 60 items that measure personality characteristics across five factors: (1) extraversion, (2) emotional instability, (3) openness, (4) integrity, and (5) agreeableness. Responses are given using a 7-point Likert scale to gauge the respondent’s level of agreement with/applicability of each statement. The Big Five Scale has been validated among a cohort of 583 Japanese university students to have high internal consistency and high concurrent and convergent validity on all five factors [41].

#### 2.2.3. Health Literacy

Health literacy, i.e., the participants’ capacity to access, understand, and utilize health-related information, was assessed using the Japanese version of the European Health Literacy Survey Questionnaire 47 (HLS-EU-Q47) [42]. The HLS-EU-Q47 was developed to comprehensively measure health literacy within a population [43] over three sub-indices, the health care health literacy index (HC-HL), disease prevention health literacy index (DP-HL), and health promotion health literacy index (HP-HL), and an overall general health literacy index (GEN-HL), which includes scores of all items. The five response options to the items gauge the level of difficulty or applicability (1 = very easy, 2 = somewhat easy, 3 = somewhat difficult, 4 = very difficult, and 5 = don’t know/not applicable). This instrument been widely used to measure health literacy internationally, including comparative studies in Asia [44]. The validity of the Japanese version of the HLS-EU-Q47 is supported by Cronbach’s alpha coefficients of 0.97, 0.92, 0.93, and 0.94 for the GEN-HL, HC-HL, DP-HL, and HP-HL indices, respectively [42].

#### 2.2.4. Self-Management

To gauge students’ abilities in managing their own behaviors and responsibilities, a validated self-management assessment tool was applied. The Self-Management Skill Scale (SMS) [45], consisting of 10 items, was used to measure the students’ self-management skills. Responses are made on a 4-point Likert scale indicating the level of applicability of the items. In this scale, higher scores indicate higher levels of self-management ability. The SMS has been validated for reliability (Cronbach’s alpha coefficient of 0.75) and showed a positive correlation with smoking cessation behavior [45].

#### 2.2.5. Exercise Habit

The shortened Japanese version of an established assessment tool, the Exercise Benefits/Barriers Scale (EBBS) [46], was used to gain insights into students’ exercise habits, focusing specifically on their perceptions of exercise benefits and barriers [47]. The reliability and validity of the original EBBS have been confirmed with Cronbach’s alpha coefficients of 0.952, 0.953, and 0.886 for the total scale, benefits scale, and barriers scale, respectively [46]. The shortened, Japanese version, the Perceived Benefits and Barriers to Exercise Scale, consists of 20 items (10 pertaining to benefits and 10 to barriers). The scale allows for the assessment of self-awareness of various factors over five benefit (physical benefit, psychological benefit, social benefit, weight management, self-improvement) and five barrier subcategories (discomfort, lack of motivation, lack of time, lack of social support, poor physical environment) [47]. Responses are given on a 5-point scale indicating level of agreement, with 1 indicating “do not agree at all” and 5 indicating “completely agree”, and higher scores indicate a stronger recognition of both health-promoting and inhibiting factors.

#### 2.2.6. Cognitive Deliberativeness and Impulsiveness

Cognitive characteristics, which reflect how individuals process information and make decisions, were investigated in this study using the Cognitive Deliberativeness-Impulsiveness Scale [48]. This Japanese scale is composed of 10 items and measures the cognitive tendency toward deliberate versus impulsive behavior with regard to decision-making. Responses for each item are made on a 4-point Likert scale indicating level of agreement with the items. This scale has been shown to have a high level of internal consistency (Cronbach’s alpha = 0.75) among a cohort of university students [48].

### 2.3. Statistical Analysis

Participant characteristics were compared by sex using an unpaired *t*-test, with adjustment for confounding factors. Partial correlation coefficients were calculated for each subscale of the MCQ-30 and the other scales, with sex as a control variable. Multiple regression analysis using the stepwise method was performed with each subscale of the MCQ-30 as the dependent variable and each of the other items as the independent variable to examine the factors associated with the MCQ-30. The significance level was set at 5%. Data are shown as mean ± SD. Statistical analyses were performed using SPSS Statistics Ver. 28 for Windows (IBM, Armonk, NY, USA). G*Power (version 3.1.9.7 for Windows, RRID, Düsseldorf, Germany) was used to calculate the required sample size for a multiple regression analysis, targeting 95% power, a 5% alpha error, and a medium effect size. The minimum required sample size was determined to be 93.

## 3. Results

### 3.1. Participant Characteristics and Survey Results

From 236 eligible students, 195 (82.6% recovery rate) responded to the survey and were included in the analyses. Of the 193 students included, 95 were female and 98 were male, with 116 in the PT department and the remaining 77 in the OT department. With the minimum required sample size determined to be 93, our final sample size of 193 was more than adequate, providing ample statistical power to detect significant effects. Table 1 shows each item as characteristics of the participants. The MCQ-30 results revealed no significant differences in scores between male and female participants across all subscales. Of the other scales, however, three items showed differences by sex. Analysis of the Big Five Personality traits indicated a significant sex difference in openness, with males scoring higher than females (53.4 ± 10.9 vs. 47.8 ± 8.5, *p* < 0.001, Cohen’s d = 0.57). This represents a medium effect size. Other personality traits, such as extraversion (51.9 ± 11.3), emotional instability (54.8 ± 12.2), integrity (45.0 ± 8.0), and harmony (53.3 ± 9.0), showed no significant differences between males and females. Significant sex differences were found in participants’ perceived benefits and barriers to exercise. Males reported significantly higher perceived benefits of exercise compared to females (40.3 ± 8.1 vs. 38.1 ± 6.7, *p* = 0.043, Cohen’s d = 0.30), while females reported higher perceived barriers to exercise than males (26.3 ± 7.1 vs. 24.0 ± 8.1, *p* = 0.045, Cohen’s d = 0.30). These differences represent a small-to-medium effect size, indicating moderate distinctions between sexes in attitudes toward exercise. These findings demonstrate that while, overall, personality traits and health literacy did not significantly vary by sex, males exhibited greater openness, and differing perceptions of exercise-related benefits and barriers were observed between the male and female students.

### 3.2. Correlation Analysis

Table 2 shows the partial correlation coefficients between each subscale of the MCQ-30 and the other scales. “Cognitive confidence” in the MCQ-30 was significantly correlated with “emotional instability” on the Big Five Scale (r = 0.49), SMS (r = 0.47), and the Cognitive Familiarity-Impulsiveness Scale (r = 0.40). “Positive beliefs” was significantly correlated with “emotional instability” on the Big Five Scale (r = 0.49), HLS-EU-Q47 DP-HL (r = −0.27), the SMS (r = 0.33), and the Cognitive Deliberativeness-Impulsiveness Scale (r = 0.52). “Cognitive self-consciousness” was significantly correlated with “emotional instability” (r = 0.58) and “openness” (r = 0.44) on the Big Five scale, the SMS (r = 0.53), and the Cognitive Deliberativeness-Impulsiveness Scale (r = 0.50). “Need to control thoughts” and “uncontrollability and danger” were significantly correlated with “extraversion” (r = −0.35), “emotional instability” (r = 0.61) and “openness” on the Big Five Scale, the SMS (r = 0.55), and the Cognitive Deliberativeness-Impulsiveness Scale (r = 0.35). A negative tendency in the “need to control thoughts” was significantly correlated with “emotional instability” (r = 0.54) and “openness” on the Big Five scale (r = 0.35), the SMS (r = 0.53), and the Cognitive Deliberativeness-Impulsiveness Scale (r = 0.42).

### 3.3. Multiple Regression Analysis

Table 3 shows the results of multiple regression analysis with the MCQ-30 “cognitive confidence” and “uncontrollability and danger” score as the dependent variable and each scale as the independent variables. The items extracted as factors influencing “cognitive confidence” were “emotional instability” on the Big Five Scale (β = 0.189), the benefits sub-scale of Exercise Benefits/Barriers Scale (β = 0.448), and the Cognitive Deliberativeness-Impulsiveness Scale (β = −0.17). The items extracted as factors influencing “uncontrollability and danger” were “emotional instability” (β = 0.189), “emotional instability” (β = 0.305), and “harmony” (β = −0.162) on the Big Five Scale. While, for the item of “emotional instability”, the data were not extracted for males (β = 0.211), the data were extracted for females (β = 0.423).

## 4. Discussion

In this study, we aimed to explore the relationship between metacognition (cognitive confidence, positive beliefs, cognitive self-consciousness, etc.), personality traits, and various factors impacting metal health to better support the well-being of students in the broader medical field throughout their education. The results of this study suggest a significant relationship between metacognition and various personality traits and characteristics among PT and OT students. The metacognitive subscales, particularly “cognitive confidence”, were found to be associated with personality traits such as “emotional instability”, as well as factors like the perceived benefits of exercise and cognitive thinking (cognitive deliberateness and impulsiveness).

One of the key findings of this study is the strong association between the metacognition “cognitive confidence” and “emotional instability”. This finding implies that students with lower cognitive self-confidence may experience higher levels of emotional instability. Multivariate analysis also showed that “emotional instability” was extracted as a factor associated with the metacognition “uncontrollability and danger” in females, although it was not extracted in males. This indicates the vulnerability of female students to mental health challenges. Emotional instability, a trait of neuroticism, is associated with other negative emotions such as anxiety, depression, moodiness, and irritability [49]. A study of Malay medical students by Yusoff and colleagues demonstrated that those who showed high neuroticism traits were more likely to develop psychological distress, being more vulnerable to negative feelings and being less able to cope with stress [50]. Interestingly, a study of Egyptian medical students found that female students scored higher than their male peers on the neuroticism scale [51]. Taken together, these findings suggest that identifying those vulnerable to emotional instability and finding metacognitive interventions to address the issue could be a crucial component to improve mental health among students in the medical field.

The study also revealed a significant connection between metacognition and cognitive thinking in terms of deliberativeness and impulsivity. “Cognitive confidence” and “positive beliefs”, two metacognitive subscales, were positively correlated with impulsivity, indicating that individuals with lower cognitive self-confidence and more positive beliefs about worries and anxiety tend to exhibit impulsiveness in their decision-making. This finding is in line with a number of studies that explore the relationship between impulsivity and metacognition [52,53,54]. Impulsivity has been shown to be associated with unhealthy behavioral and lifestyle choices, like aggressive tendencies [55] and addictions [54]. Thus, for students in the medical field and adolescents in general, interventions to build cognitive self-confidence and reduce anxiety could help to address such destructive tendencies.

Health literacy, exercise habits (perceived benefits and barriers), and “openness”, a personality trait from the Big Five Scale, were also identified as being associated with metacognition in this study. Health literacy was negatively correlated with “positive beliefs”, indicating that individuals with higher health literacy may be less prone to anxiety. This has been particularly pertinent during the COVID-19 pandemic, where increased infectious-disease-specific health literacy seemed to have a correlation with reduced anxiety [56]. Indeed, among students studying among the various medical-related fields in Japan and elsewhere, health literacy seems to have had a positive effect on motivation [57,58,59].

Exercise habits, specifically, the perceived benefits of exercise, were positively associated with metacognition, highlighting, as has long been known, the potential benefits of physical activity on cognitive processes [60,61,62]. Thus, interventions involving physical exercise may be helpful to address issues of mental health and improve cognitive processes. Indeed, some studies in medical students have shown the benefits of aerobic exercise [63] and yoga on cognitive function [64]. Additionally, in our study, males reported higher benefits from exercise habits compared to females, while females reported higher inhibitory factors (barriers) to exercise compared to males. Metacognitive processes, like cognitive self-awareness, could influence how individuals perceive and address barriers to exercise, as those with stronger metacognitive skills may be better at identifying and challenging negative beliefs. Conversely, individuals with weaker metacognitive abilities may struggle to reframe these barriers, potentially making it harder for them to engage in exercise. Efforts to promote sport and physical activity among female students could be beneficial for promoting their mental well-being. In stark contrast, the COVID-19-related lockdown policies adopted in many countries, which included gym closures, stay-at-home orders, and the large-scale uptake of online learning, had a very negative impact on physical and mental health [65,66,67]. Policy makers and educators should be aware of the importance of physical activity in the physical and mental well-being of students.

Finally, the personality trait of “openness” on the Big Five Scale was linked to several metacognitive subscales, suggesting that individuals who are more open to new experiences may exhibit varying metacognitive patterns. We found that males scored significantly higher in “openness” compared to females. Openness has been shown to correlate somewhat with creativity and intellect [68,69]; however, its complex interaction with metacognition has also been observed in previous studies, being described as a “double-edged sword” that “predisposes individuals to feel the good and the bad more deeply” [59]. Generally, however, openness, together with other traits such as conscientiousness and extraversion, is an important component of the personality of those working in the medical professions [70,71].

### Limitations

It is essential to acknowledge some limitations of this study. Firstly, this study was conducted with a specific population of Japanese students from a single institution studying physical and occupational therapy. This limits the generalizability of the findings to students of other cultures or in different medical fields, which have distinct academic demands and stressors. Secondly, the use of a cross-sectional design restricts its ability to establish causal relationships among metacognition, personality traits, and the other factors. Future longitudinal studies would be useful for exploring causality and the effectiveness of metacognitive interventions targeting emotional instability and cognitive thinking. In this study, we examined various personality traits, metacognition, and related factors; however, we may have overlooked additional variables, such as external stressors or significant life events, including the experience of the COVID-19 pandemic, which could also influence the observed relationships. Finally, the study relied on self-administered questionnaires for data collection, which can introduce response bias and/or social desirability bias. Participants may not always provide accurate or honest responses, especially when discussing sensitive topics like mental health and personality traits. To mitigate this limitation in future research, a mixed-methods approach could be employed, combining self-report measures with qualitative interviews or focus groups to gain deeper insights and cross-validate the findings. However, the findings of the study were in line with previous research and contribute to a growing body of knowledge on mental health among students within healthcare-related professions and offer practical insights for educators, administrators, and policymakers seeking to improve the well-being of students and, by extension, future healthcare professionals.

## 5. Conclusions

In conclusion, this study provides valuable insights into the relationship between metacognition, personality traits, and various factors influencing mental health among PT and OT students. Our findings suggest the vulnerability of students, including those studying health-related fields, to mental health challenges, and indicate that female students might be at particular risk. This research successfully addressed our objective to explore how metacognitive processes and personality traits, such as emotional instability and cognitive self-confidence, interact with factors that might impact mental well-being. By understanding these relationships, we can hopefully better support students throughout their educational journey. The findings of this study should encourage educational institutions to prioritize mental health awareness and provide resources for students to cope with stress and emotional instability. University programs, particularly those within the medical field, may consider incorporating educational elements or activities that boost cognitive self-confidence and emotional resilience into their curricula such as cognitive behavioral techniques, mentorship and peer support initiatives, mindfulness, and stress-management training. This could better prepare students for navigating the demands of their future professions in healthcare. The findings highlight the importance of considering individual characteristics when designing interventions to support students’ mental well-being. Specifically, the study emphasizes the potential benefits of improving cognitive self-confidence and promoting physical activity as strategies for enhancing metacognition and, consequently, mental health among college students.

## Figures and Tables

**Table 1:** Participant characteristics and survey results by sex.

	Total	Male	Female		Effect Size
	(*n* = 193)	(*n* = 98)	(*n* = 95)	*p*-Value	(Cohen’s d)
Age (years)	20.3 ± 3.4	20.5 ± 3.5	20.2 ± 3.2	0.830	0.09
BMI (kg/m^2^)	22.7 ± 4.7	23.3 ± 5.4	22.1 ± 3.6	0.080	0.26
MCQ-30 (scores)					
Cognitive confidence	16.3 ± 2.9	16.1 ± 2.8	16.5 ± 3.0	0.153	0.14
Positive beliefs	16.0 ± 3.3	16.0 ± 3.4	16.0 ± 3.3	0.838	0.00
Cognitive self-consciousness	14.4 ± 3.3	14.4 ± 3.2	14.4 ± 3.5	0.883	0.00
Uncontrollability and danger	14.6 ± 3.8	14.8 ± 3.7	14.4 ± 3.8	0.252	0.11
Need to control thoughts	14.3 ± 3.8	14.6 ± 3.7	14.1 ± 3.6	0.135	0.14
Big Five Scale (scores)					
Extraversion	51.9 ± 11.3	52.1 ± 11.8	51.8 ± 9.4	0.699	0.03
Emotional instability	54.8 ± 12.2	53.7 ± 11.9	56.4 ± 11.1	0.230	0.23
Openness	50.4 ± 10.8	53.4 ± 10.9	47.8 ± 8.5	0.000 **	0.57
Integrity	45.0 ± 8.0	45.6 ± 7.3	44.9 ± 7.3	0.551	0.10
Harmony	53.3 ± 9.0	53.9 ± 8.8	53.2 ± 7.5	0.526	0.09
HLS-EU-Q47 GEN-HL (scores)	22.5 ± 6.4	22.6 ± 6.2	22.3 ± 6.1	0.602	0.07
HLS-EU-Q47 HC-HL (scores)	28.7 ± 8.3	28.3 ± 8.1	29.5 ± 8.1	0.208	0.15
HLS-EU-Q47 DP-HL (scores)	19.2 ± 5.8	19.7 ± 6.1	18.9 ± 5.2	0.478	0.14
HLS-EU-Q47 HP-HL (scores)	26.9 ± 6.6	26.7 ± 6.2	27.5 ± 6.5	0.177	0.13
Self-Management Skill Scale (scores)	27.6 ± 2.8	27.6 ± 3.1	27.5 ± 2.5	0.877	0.04
Perceived Benefits and Barriers to Exercise Scale: Benefits (scores)	39.2 ± 7.5	40.3 ± 8.1	38.1 ± 6.7	0.043 *	0.30
Perceived Benefits and Barriers to Exercise Scale: Barriers (scores)	25.1 ± 7.7	24.0 ± 8.1	26.3 ± 7.1	0.045 *	0.30
Cognitive Deliberativeness-Impulsiveness Scale (scores)	27.9 ± 4.6	28.0 ± 4.8	27.8 ± 4.5	0.715	0.04

Notes. Mean ± SD. Unpaired t-test. Effect size: Cohen’s d. *: *p* < 0.05; **: *p* < 0.01; BMI: Body Mass Index; MCQ-30: Metacognitive Questionnaire 30; HLS-EU-Q47: European Health Literacy Survey Questionnaire 47.

**Table 2:** Relationship between the MCQ30 and other variables: partial correlation analysis.

	Cognitive Confidence		Positive Beliefs		Cognitive Self-Consciousness		Uncontrollability and Danger		Need to Control Thoughts	
	PCC	*p*	PCC	*p*	PCC	*p*	PCC	*p*	PCC	*p*
Big Five Scale (scores)										
Extraversion	−0.09	0.533	−0.14	0.299	−0.10	0.476	−0.35	0.010 *	−0.02	0.907
Emotional instability	0.49	0.000	0.49	0.000	0.58	0.000 **	0.61	0.000 **	0.54	0.000 **
Openness	0.08	0.587	0.19	0.173	0.44	0.001 **	0.29	0.036 *	0.35	0.011 *
Integrity	−0.04	0.766	0.06	0.679	−0.08	0.550	−0.22	0.111	−0.10	0.479
Harmony	−0.11	0.435	0.10	0.478	0.10	0.489	−0.16	0.256	0.08	0.551
HLS-EU-Q47 GEN-HL (scores)	0.24	0.078	−0.20	0.15	0.12	0.389	0.04	0.789	−0.02	0.883
HLS-EU-Q47 HC-HL (scores)	0.16	0.236	−0.19	0.171	0.09	0.530	0.00	0.991	−0.04	0.792
HLS-EU-Q47 DP-HL (scores)	0.19	0.176	−0.27	0.046	−0.08	0.570	−0.09	0.502	−0.13	0.335
HLS-EU-Q47 HP-HL (scores)	0.27	0.050	−0.04	0.796	0.27	0.050	0.11	0.419	0.07	0.612
Self-Management Skill Scale (scores)	0.47	0.000	0.33	0.017	0.53	0.000 **	0.55	0.000 **	0.53	0.000 **
Exercise Benefits/Barriers Scale: Benefits (scores)	−0.189	0.170	−0.04	0.764	−0.03	0.834	−0.10	0.495	0.02	0.901
Exercise Benefits/Barriers Scale: Barriers (scores)	0.05	0.703	0.01	0.961	−0.05	0.730	0.13	0.351	0.03	0.825
Cognitive Deliberativeness-Impulsiveness Scale (scores)	0.40	0.002	0.52	0.000	0.50	0.000 **	0.35	0.010 *	0.42	0.002 **

Notes. Controlled variable = sex; PCC = partial correlation coefficient; * *p <* 0.05; ** *p <* 0.01.

**Table 3:** Factors associated with the MCQ-30 “cognitive confidence” and “uncontrollability and danger” scores: stepwise selection.

	β	95%CI (Lower Limit–Upper Limit)	*p*-Value
Cognitive Confidence (*n* = 193)			
Big Five Scale “emotional instability”	0.189	(0.005–0.089)	0.028 *
Exercise Benefits/Barriers Scale: benefits (scores)	0.448	(−0.128–−0.005)	0.034 *
Cognitive Deliberativeness-Impulsiveness Scale (scores)	−0.17	(0.172–0.360)	<0.01 **
Uncontrollability and danger (*n* = 193)			
Big Five Scale “extraversion”	−0.172	(−0.116–−0.003)	0.038 *
Big Five Scale “emotional instability”	0.305	(0.047–0.153)	<0.01 **
Big Five Scale “harmony”	−0.162	(−0.142–−0.009)	0.026
Male (*n* = 98)			
Big Five Scale “emotional instability”	0.211	(0.513–1.949)	0.100
Female (*n* = 95)			
Big Five Scale “emotional instability”	0.423	(0.067–0.248)	<0.01 **

Notes. Adjusted R2: 0.276. *: *p* < 0.05; **: *p* < 0.01. β: standardized regression coefficient; 95%CI: 95% Confidence Interval.

## Data Availability

The data presented in this study are available on request from the corresponding author. The data are not publicly available due to privacy restrictions.
